# RECIST measurements in cancer treatment: is there a role for physician assistants? - A pilot study

**DOI:** 10.1186/1470-7330-14-12

**Published:** 2014-04-22

**Authors:** Anna M Sailer, Dave CE Douwes, Vincent C Cappendijk, Frans C Bakers, Bart AJM Wagemans, Joachim E Wildberger, Alfons G Kessels, Regina GH Beets-Tan

**Affiliations:** 1 Department of Radiology, Maastricht University Medical Centre (MUMC), P. Debyelaan 25, 6229 HX Maastricht The Netherlands; 2 Siemens Netherlands, Sector Healthcare, Clinical Education, The Hague, The Netherlands; 3 Department of Radiology, Jeroen Bosch Hospital, ‘s Hertogenbosch, The Netherlands; 4 GROW – School for Oncology and Developmental Biology, Maastricht University, Maastricht, The Netherlands; 5 Department of Clinical Epidemiology and Medical Technical Assessment, Maastricht University Medical Centre, Maastricht, The Netherlands

**Keywords:** RECIST 1.1, Target lesion measurement, Interobserver variability, Radiology physician assistant, Paramedics, Radiological unit workflow

## Abstract

**Background:**

Decision making in cancer treatment is influenced by standardized RECIST measurements which are subjective to interobserver variability. Aim of this pilot study was to evaluate whether it is feasible to transfer the radiologist’s task of RECIST measurements to a trained radiology physician assistant and whether this influences diagnostic performance.

**Methods:**

177 lesions in twenty patients were measured on baseline and two follow-up CTs using RECIST 1.1: Arm A according to routine clinical practice where various radiologists read scans of the referred patients. Arm B according to the experimental setting where a radiology physician assistant performed RECIST measurements of target lesions defined by the radiologists on baseline scans. Performance and agreement were compared between groups.

**Results:**

Standard deviation between lesion measurements of arm A and B was four millimeters. Interobserver agreement comparing response category classification was substantial, ĸ = 0.77 (95% CI: 0.66 - 0.87). Sensitivity and specificity for the radiology physician assistant for assessing progressive disease were 100% (95% CI: 61% - 100%) and 94% (95% CI: 81% - 98%) respectively.

**Conclusion:**

RECIST measurements performed by a paramedic are a feasible alternative to standard practice. This could impact the workflow of radiological units, opening ways to re-assigning radiologists’ important, standardized but time consuming tasks to paramedics.

## Background

Evaluation of tumor burden with imaging plays an important role in the clinical assessment of response to cancer therapy and subsequent treatment decision as well as in clinical trials to evaluate the effect of new drugs. Assessment of disease progression will influence the decision on whether or not the treatment should be discontinued. Reduction in tumor size and volume is a marker of treatment effect in early phase therapeutic trials. Response assessment by imaging methods hence needs to be highly reproducible and accurate.

Guidelines recommend lesion size measurements following standardized methods such as WHO criteria [1] and RECIST 1.0 [2]. Recently the RECIST committee introduced a new version of Response Evaluation Criteria In Solid Tumors: RECIST 1.1 [3,4]. Following these criteria, a total number of up to five target lesions are chosen to follow-up during and after therapy. Up to two lesions per organ, with a minimum long axis of diameter ten millimeters in solid lesions and fifteen millimeters in lymph nodes in an axial plane should be identified and measurements compared to prior assessment [5,6].

Solid tumor assessment following the RECIST criteria is now standard both in clinical practice and trial settings, mostly practiced on CT chest and/or CT abdomen. In trial settings, measurements are performed by trial radiologists in order to assure consistency. Contrary to that, in daily clinical routine, scans of the individual patient are often evaluated by several radiologists at different time points along the treatment and only very rarely handled by the same reader. Previous studies have shown inaccuracy in assessment due to considerable interobserver variability in measurements [7-13]. Inconsistency in measurements can lead to incorrect interpretation in tumor response. A prior study advocated a restriction in the number of radiologists analyzing RECIST scans in order to prevent interobserver variation and misinterpretation [8]. Our hypothesis is that RECIST 1.1 measurements of lesions on response evaluation CTs – a highly relevant but time consuming task of the radiologist - are as accurate and probably more consistent in the hands of specialized radiology physician assistants as in that of several radiologists handling one individual patient’s scans along his treatment. If this can be confirmed, transferring RECIST routine measurements to radiology physician assistants could not only potentially increase cost efficiency but also free the time of radiologists whose responsibilities as sparring partner in the multidisciplinary cancer management team are increasing.

The aim of this study was to compare the assessment of tumor burden performed by various board certified radiologists (the common clinical setting of RECIST CT evaluation) to the assessment performed by a trained CT radiology physician assistant.

## Methods

This retrospective study was approved by the local ethics committee (Medisch-ethische toetsingscommissie (METC aZM/UM)) as a quality control pilot study without further need for informed consent.

The study group consisted of twenty patients. Inclusion criteria were: (1) patients with proven solid tumors, (2) treatment with anti-neoplastic therapy, (3) RECIST 1.1 as principle evaluation criteria of tumor response, (4) CT baseline and follow-up studies performed between 2009 and 2011.

**Figure 1:**
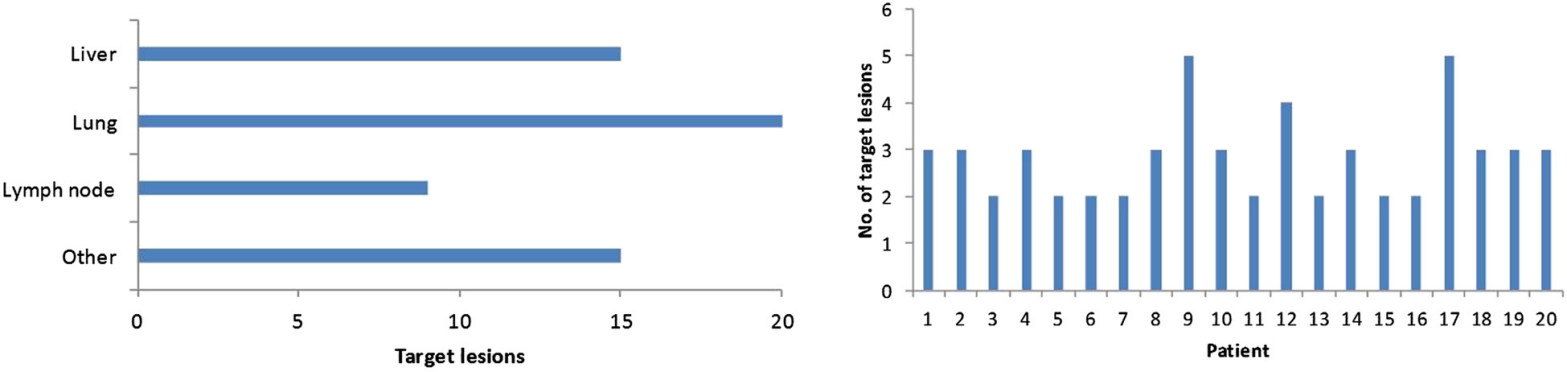
Type of target lesions in group of patients (left image) and number of selected target lesions per patient (right image).

Eight patients had colorectal cancer, seven patients had renal cancer, three patients had breast cancer, one had an ovary carcinoma and one had pancreatic cancer. The mean age of the patients was 61 ± 10 years and the mean follow-up time during the study was seven months. Figure 1 gives detailed overview on target lesions.

CT scans were performed with the same protocol, contrast-enhancement, five millimeter slice thickness and were evaluated in standard lung, abdominal and liver window.

In all patients CTs of the chest and/or abdomen were performed on a baseline before therapy started as well as two times for follow-up during and after anti-neoplastic therapies.

### Image evaluation

**Figure 2:**
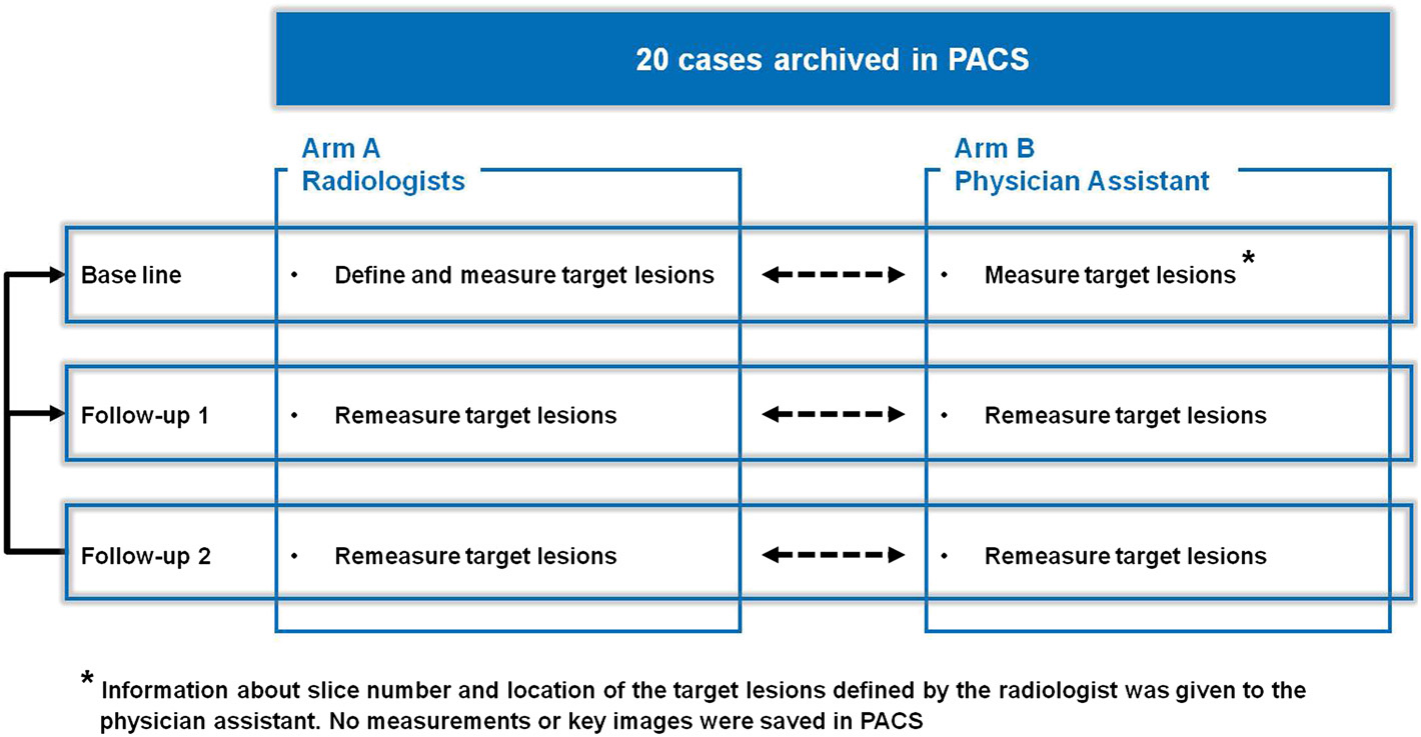
Study design.

Study consisted of two evaluation arms: Arm A presenting the standard clinical practice where various board certified radiologists read the scans of the patients ad random. As in usual practice none of them attempted to only read the follow-up CTs of those patients for whom they have evaluated the baseline CT. In other words one patient’s set of CTs could have been evaluated by several radiologists, but all were specialized in abdominal imaging. The radiologist who evaluated the baseline CT defined target lesions following the RECIST 1.1 criteria. Arm B: a radiology physician assistant with CT experience of more than ten years read and assessed all follow-up CT scans. The target lesions at baseline CT were indicated by the radiologists but their RECIST measurement not revealed to the assistant. The physician assistant re-measured the lesions both on baseline and follow-up CTs. Figure 2 shows a scheme of the study design.

Fifty-nine target lesions were defined by the radiologists. A total of 177 target lesion measurements in twenty patients were performed by both groups during the study period.

Measurements of each lesion on each time point were compared between the two study arms. Following the RECIST 1.1 criteria, response categories consisting of stable disease (SD), complete response (CR), partial response (PR) and progressive disease (PD) were also compared between the arm A and B. This was performed on two time points, at follow-up 1 and 2.

All CT images where archived and reviewed in the institution’s Picture Archiving and Communication System PACS, (Carestream Health Inc., Rochester, NY, USA). Tumor size was measured by electronic calipers.

### Statistical analysis

Interclass correlation coefficient was used to compare the measurements of the physician assistant to standard clinical practice and prediction intervals were constructed by means of a linear regression model. A Bland-Altman plot was performed for illustration of differences in measurements. Taken tumor assessment performed by the radiologists as the standard of reference, sensitivity and specificity of detecting progressive disease by the assistant were analyzed. Kappa statistics was applied to evaluate agreement concerning response category classification between the physician assistant and standard clinical practice. Statistical analyses were performed using SPSS Statistics 20.0 (SPSS, Chicago IL, USA).

## Results

**Figure 3:**
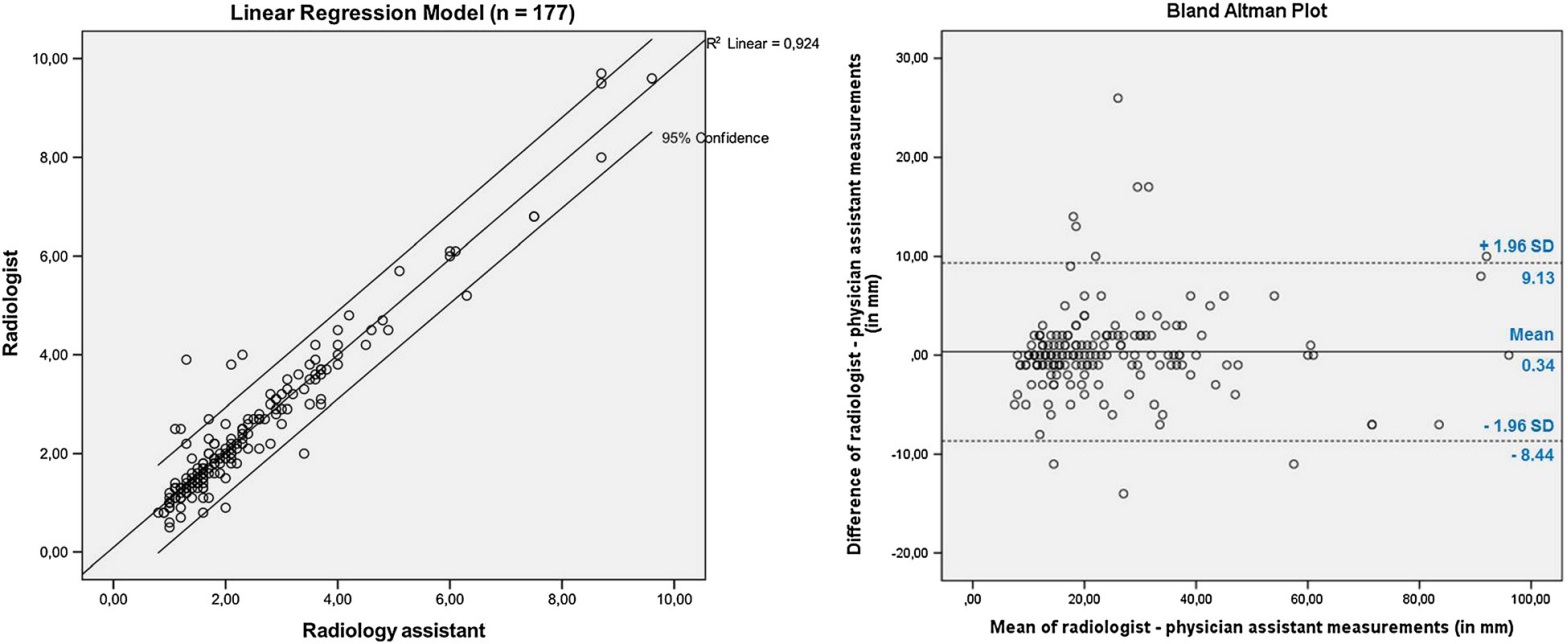
**Prediction intervals by linear regression model (left) and Bland-Altman plot (right).** N = 177.

Comparison of the 177 lesion measurements between the two study arms by means of linear regression model and Bland-Altman plotting is shown in Figure 3.

Taken the radiologists’ measurements as the reference standard, the linear regression model shows the prediction of the radiologists’ results for the measurement performed by the assistant. Regression coefficient was R^2^ = 0.924. Interclass correlation coefficient between arm A and B was 0,961. Mean lesion size were twenty-four millimeters for both arms. Standard deviation between arm A and B for all 177 lesion measurements was four millimeters, which was 18% of the mean lesion size. Considering only differences between the radiologist and the assistant’s measurement greater than two millimeters, there was a concordance in assessment of 70% of all measurements.

According to RECIST 1.1 criteria, the measurements of arms A and B were used to judge tumor burden and classify into response categories. Interobserver agreement concerning category classification between arm A and B was substantial, ĸ = 0.77 (95% CI: 0.66 - 0.87). The physician assistant did not underestimate response category. In four cases there was an overestimation of response category by the assistant. Table 1 shows the agreement concerning progressive disease, partial response, stable disease and complete response for the two study arms.

**Table 1:** Comparison of the two arms regarding tumor responses at follow-up 1 and follow-up 2 according to RECIST 1.1 measurements

		**Arm A: radiologists**				
		**CR**	**PR**	**SD**	**PD**	**Total**
**Arm B:**	**CR**	0				0
**Physician assistant**	**PR**		3			3
**Assistant**	**SD**		2	27		29
	**PD**			2	6	8
	**Total**	0	5	29	6	40

Notes: CR: Complete Response, PR: Partial Response, SD: Stable Disease, PD: Progressive Disease.

Sensitivity for detecting progressive disease (PD) for the physician assistant (B) was 100% (95% CI: 61% - 100%), specificity was 94% (95% CI: 81% - 98%), respectively.

## Discussion

Response assessment by imaging before and during cancer therapy influences clinical decision making on whether or not therapy should be continued. The role of the radiologist is consolidated and his task is threefold.

He needs to confirm the presence or absence of new suspicious lesions, rule out any acute threatening conditions such as impeding fracture or spinal cord compression and assess the overall tumor burden and predict whether or not there is a response by measuring changes in tumor size during therapy. For the latter, RECIST 1.1 criteria are widely adopted in standard clinical practice. Most of the RECIST assessment is based on CT images. These target lesion measurements need to be accurate and reproducible. Such measurements are time consuming, especially because the baseline or previous follow-up CT scans of the individual patient are not necessarily read by one and the same radiologist and are often handled by several radiologists along the treatment. Therefore these measurements are known to be prone to significant interobserver variability [7-13]. This matter of lack of consistency has already successfully been addressed in trial settings, where measurements are widely performed by trial radiologists. However, in routine clinical practice, oncologists are often confronted with inconsistent measurements of lesions at consecutive follow-up scans.

Therefore there is a need to improve the assessment of treatment response evaluation by imaging in clinical routine. Our hypothesis is that RECIST 1.1 routine measurements of lesions on consecutive CTs can be as accurate and probably more consistent in the hands of dedicated radiology physician assistants, who read all surveillance CTs of one individual patient, as in that of several radiologists handling one individual patient’s scans along his treatment.

We believe that transferring of these routine tasks from the radiologists to dedicated and trained radiology physician assistants will reduce interobserver variability, improve consistency and contribute to a more time and cost efficient diagnostic workflow. Introducing paramedic assistants supporting the daily task of physicians is a well accepted phenomenon at many clinical units. Multidisciplinary teams have recognized the benefit in time and cost-efficiency if nurse/physician assistants support their daily practice in outpatient clinics and on hospital wards. Although so far radiologists have been more reluctant to involve paramedics in the diagnostic making process, it is inevitable that reorganization of radiological units needs to happen should the high quality of care be met without further increasing the costs. In current oncological radiology practice there is a huge demand on the radiologists involved in multidisciplinary decision making. His attendance is expected at multidisciplinary cancer meetings and his responsibilities are extending beyond reading and reporting images. Interaction with clinicians is as important as evaluation of images, but hiring more radiologists to cover all the workload would not be a realistic, certainly an expensive solution. Thus it will be necessary to reorganize and to find ways to free the radiologists’ time without compromising diagnostic outcome.

To our knowledge this is the first study evaluating whether transferring the task of RECIST lesion measurements by radiologists to CT experienced radiology physician assistants could affect diagnostic performance and outcome.

The results of our pilot study are promising showing high agreement between the radiologists’ and the radiology physician assistant’s performance. Sensitivity for detecting progressive disease (PD) for the assistant was 100% and specificity was 94% respectively. There was no underestimation of response category by the assistant. According to RECIST guidelines, three measurements were performed within each patient at different time points implying serial dependency of the data. This is a limitation due to the study design. Furthermore, this pilot study is based on a restricted number of patients and observations, which is why outcome should be evaluated in a larger setting. However, results show that involving paramedic assistants in the diagnostic workflow does not necessarily compromise outcome, if this happens with well-trained physician assistants and under close monitoring by a radiologist.

In our study, target lesions were defined by radiologists on the baseline scan and information was passed to the radiology physician assistant including key images, image number and lesion location and measurements. A potential detection of new lesions by the radiologist assistant was not evaluated. In this way the diagnostic decision making remained the responsibility of the radiologist. We believe that such an approach guarantees the high quality of standard care. In this respect we also think that the paramedic should work side by side with the radiologist in the reporting unit, so that – whenever necessary - the radiologist, who is responsible for authorizing the report, is at easy reach for consultation.

We recognize that physician assistants are not trained to diagnose. This is a responsibility that should remain with radiologists. In our opinion, definition of target lesions, detection of new lesions as well as critical lesions should be part of the diagnostic task assigned to the radiologist, in order to assure reliability. Hence our reading design. We are aware that in this way, the radiologist’s time will still be consumed by reviewing and approving the assistant’s findings. In the first phase of implementation of the new workflow, additional supervision (and education) of the assistant will require an additional time investment from the radiologist and it might be that in this phase the radiologist would have been more efficient if he would do the evaluation solely. However, after a learning curve and with increasing experience of a well-trained assistant, actual time needed for supervision is expected to rapidly decrease. Based on our experience, lesion measurements and comparison of each lesion measurement with that of previous scans was in fact the most time consuming part of RECIST evaluation. Re-assignment of this task in routine practice to a trained physician assistant is therefore expected to be time efficient in the long run. Nevertheless, dedicated cost-effectiveness analysis of this approach has yet to happen.

## Conclusion

In conclusion, we believe that the approach of transferring target lesion measurements to radiology physician assistants could, besides reducing the measurement variability, hold benefits in terms of time efficiency and allow radiologists to focus on addressing more complex diagnostic dilemmas and on interactions with clinicians. Assuming a lower cost of radiology assistant’s manpower, this approach is also suspected to be cost-efficient. The results from our pilot study - if confirmed in further larger prospective studies - will therefore potentially impact clinical practice.

## Competing interest

We certify that there is no conflict of interest with any financial or other organization regarding the work contributing to this article.

## Authors’ contributions

AMS did acquisition of data, data analysis, literature research and drafted the manuscript. DCED and VCC did acquisition of data. FCB did acquisition of data and data analysis. BAJMW and AGK did data analysis and statistics. JEW edited the manuscript. RGHB-T drafted the concept of the study and edited the manuscript. All authors read and approved the final manuscript.
